# Impact of COVID-19-Related Social Distancing on the Incidence of Inpatient Aggression at a Public Psychiatric Hospital

**DOI:** 10.7759/cureus.49440

**Published:** 2023-11-26

**Authors:** Salman Akram, Ankur Sah Swarnakar, Madeline Lodeiro, Ahmad Jalil, Aleena Majeed, Fahad Mukhtar, Faisal Akram

**Affiliations:** 1 Psychiatry and Behavioral Sciences, Saint Elizabeths Hospital, Washington, USA; 2 Psychiatry, Saint Elizabeths Hospital, Washington, USA; 3 Internal Medicine, Mayo Hospital, Jacksonville, USA; 4 Psychiatry, Fatima Jinnah Medical University, Lahore, PAK; 5 Psychiatry and Behavioral Sciences, Sheppard Pratt Hospital, Baltimore, USA; 6 Psychiatry, Mindpath Health, Oakland, USA

**Keywords:** covid-19, physical restraints, state psychiatric hospital, social distancing, inpatient aggression

## Abstract

Objective: This study explores the impact of coronavirus disease (COVID) pandemic-related social distancing measures on the incidence of inpatient aggression at a public psychiatric hospital

Methods: Data was gathered from the hospital’s unusual incident (UI) database for the time period ranging from January 1, 2015, to December 31, 2020. Based on the implementation of major social distancing measures, March 6, 2020, was set as a cutoff time point to categorize aggressive events into pre-COVID and post-COVID groups. Data was analyzed using Chi-square tests and general linear modeling. The p-value was set at ≤0.05.

Results: After the implementation of social distancing measures, there was a decrease in the absolute number of inpatient aggressive events from 15.0/week to 12.6/week (mean difference: 2.4/week, p=0.032). However, this decrease was primarily attributable to a decrease in hospital census. There was a decrease in the proportion of seven-day and 14-day post-admission aggressive events by 5.4% and 12.1%, respectively. Concurrently, there was a 4.9% increase in recurrent aggression. Emergency psychiatric medication administration and the use of physical restraint decreased during the COVID-19 pandemic.

Conclusion: Consistent with previous results, this study reports a decrease in the incidence of inpatient aggression during the COVID-19 pandemic. Social distancing measures can be utilized as a tool to decrease the incidence of inpatient aggression and the use of physical restraints.

## Introduction

Aggression is a common occurrence in inpatient psychiatric units because it is one of the main reasons for psychiatric admission [1,2]. Prevalence estimates vary from eight to 76% and almost one in five patients admitted to acute psychiatric units may commit an act of violence [1,3]. The disproportionate percentage of aggressive acts in psychiatric units leads to a plethora of negative consequences. First, injury to patients and staff is a frequent and undesirable consequence of aggression. Victims of these events are significantly distressed, sometimes physically injured suffer from high disease rates and low work satisfaction [4-7]. Second, the psychological trauma to both the staff members and the patients is remarkable [8]. Some staff showed the intention to leave the organization, and some did not return to work after the assault leading to high staff turnover and high vacancy rates [9,10]. Aggression can also result in significant costs to mental health services [11,12].

A recent systematic review showed that risk factors of inpatient aggression can be categorized into patient, staff, and ward-related factors [1,13,14]. Younger age, schizophrenia spectrum disorders, bipolar disorders, substance use, neurocognitive disorders, delirium, and prior history of aggression are well-known patient-related risk factors [15-22]. Staff factors that are associated with aggression include male gender, job dissatisfaction, burnout, inadequate staff training, and poor collaboration between nurses and other staff members [1,23-25]. Among ward-related factors, higher patient bed occupancy rates and ward crowding are significantly associated with increased risk of inpatient aggression [1,26]. Furthermore, few or poorly organized activities in the unit, fear about the staff-patient relationship, and poor staff-patient interaction are known to increase the risk of inpatient aggression [1].

The coronavirus disease pandemic of 2019 (COVID-19) forced many changes in hospital practices to ensure adequate social distancing to prevent the spread of infection. At the study site, these changes included a reduction in hospital census, decrease in the number of admissions, separation of new admissions from other inpatients, decrease in the number of therapeutic groups, and decrease in the number of individuals in the dining area. Many of these social distancing measures such as decrease in the number of patients and decreased patient-to-patient interactions have been previously associated with a decrease in the incidence of inpatient aggression. Therefore, this study explores the impact of COVID-19-related social distancing on the incidence of inpatient aggression in locked psychiatric units.

## Materials and methods

The study protocol was approved by the Institutional Review Board of Saint Elizabeths Hospital, Washington DC. Deidentified aggregate data was collected from information available on the unusual incident (UI) database. Saint Elizabeths Hospital has implemented a UI reporting system whereby all UIs, including aggression-related UIs, are reported to hospital management to ensure adequate monitoring and maintenance of patient safety. Additional data including data on medication refusals and hospital census was requested from the data management team of hospital. Data was collected for the years 2015-2020. The sample population was limited to adult patients aged 18 years and above. The study population included forensic and civil patients identified separately in the data set based on information recorded at the time of admission. Based on the timing of the implementation of social distancing measures, March 6, 2020, was set as a cutoff time point to categorize inpatient aggressive events into pre-COVID and post-COVID groups. Chi-square tests were performed to compare the two groups. Individual aggressive events were grouped into weekly aggression rates. General linear modeling was performed to adjust for the number of patients in the unit and medication refusals. Aggressive events were further categorized into “verbal aggression,” “aggression to patient,” “aggression to staff,” “property destruction,” and “aggression to self” groups to specify the nature of aggressive events. Data regarding the severity of aggressive events was collected in the form of variables indicating if emergency psychiatric medications, physical restraint, or physical hold were utilized in the management of aggressive behavior. An aggressive event was labeled as “recurrent aggression” if an individual in care had an aggressive event within seven days of another previous aggressive event. Similarly, seven-day and 14-day post-admission aggressive events were identified by calculating the number of days between the psychiatric admission and the aggressive event. The p-value was set at ≤0.05.

## Results

**Figure 1:**
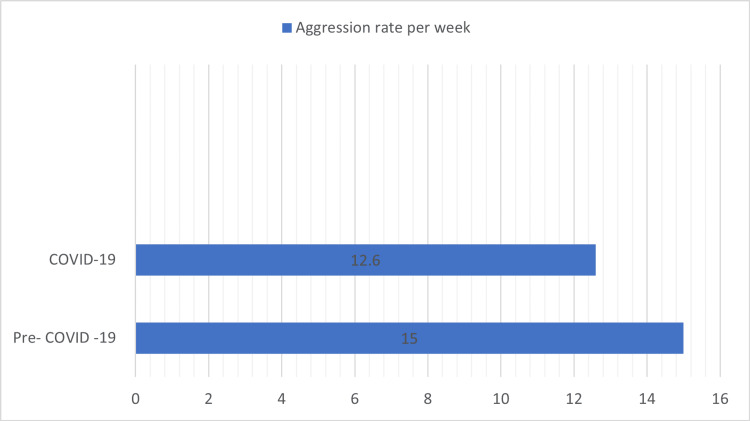
Comparison of aggression rate per week in the pre-COVID-19 and COVID-19 period. The mean difference was 2.4/week, p-value=0.032

**Figure 2:**
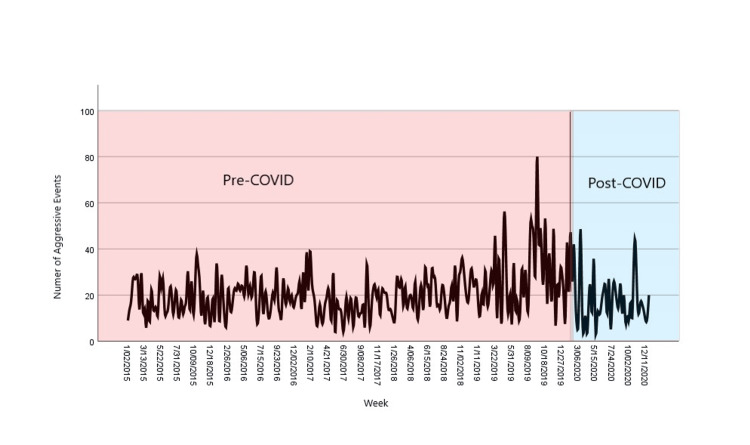
Weekly rate of aggressive events before and after COVID-19 pandemic

Table 1 shows the comparison of aggressive events between the pre-COVID and post-COVID groups. After the implementation of social distancing measures, there was a decrease in absolute number of inpatient aggressive events from 15.0/week to 12.6/week (mean difference: 2.4/week, p-value=0.032) (Figure 1). The graphical representation of the differences is shown in Figure 2. However, this decrease was primarily attributable to a decrease in hospital patient census. The weekly census-adjusted aggressive event rate in the pre-COVID group was 5.7/100 patients compared to 6.1/100 patients in the post-COVID group. Weekly aggressive event rate correlated with the average weekly patient census (p=0.015, Pearson correlation coefficient r=0.14). Subgroup analysis showed that the relationship of bed occupancy was only significant for forensic admissions (p=0.027, Pearson correlation coefficient r=0.13). The correlation between civil admissions and inpatient aggression was not significant (p=0.11). Weekly aggressive event rate was also significantly associated with the total number of medication refusals in the unit (p=0.013). A comparison of the pre-COVID and post-COVID groups showed that there was a decrease in the proportion of seven-day and 14-day post-admission aggressive events by 5.4% (p<0.001) and 12.1% (p<0.001), respectively. Concurrently, there was a 4.9% increase in recurrent aggression (p=0.016). Only 32.7% of aggressive events (compared to 46.6% in the pre-COVID group) were considered psychiatric emergencies after the implementation of social distancing measures (p<0.001). Emergency psychiatric medication usage for management of aggression decreased by 28.2% (p<0.001) during the COVID-19 pandemic. Similarly, physical hold and restraint events decreased by 13.5% (p<0.001) and 3.6% (p=0.032), respectively.

**Table 1:** A comparison of parameters of inpatient aggressive events before and after the implementation of social distancing measures during the COVID-19 pandemic Physical hold and restraint are emergency safety measures of last resort that restrict a person’s freedom to move and are used to prevent damage from an aggressive event. *denotes statistical significance OR, odds ratio; CI, confidence intervals

	Pre-COVID Group (n) (%)	Post-COVID Group (n) (%)	Total	P-Value	OR (95% CI)
N	4114	532	4646		
	88.5%	11.5%	100%		
Aggression to patients	787	98	885	0.695	0.95 (0.76-1.21)
	19.10%	18.40%	19%		
Aggression to staff	2653	347	3000	0.738	1.03 (0.85-1.25)
	65.20%	64.50%	64.60%		
Verbal aggression	135	54	189	<0.001^*^	3.3 (2.40-4.63)
	3.30%	10.20%	4.10%		
Aggression to self	313	46	359	0.399	1.14 (0.83-1.58)
	8.60%	7.60%	7.70%		
Property destruction	501	86	587	0.009^*^	1.4 (1.08-1.8)
	16.20%	12.20%	12.60%		
Psychiatric emergency	1917	174	2091	<0.001^*^	0.55 (0.46-0.67)
	46.60%	32.70%	45%		
Seven-day post-admission aggression	446	29	475	<0.001^*^	0.49 (0.33-0.74)
	11.10%	5.70%	10.50%		
14-day post-admission aggression	742	30	772	<0.001^*^	0.28 (0.19-0.40)
	18%	5.90%	17.00%		
Recurrent aggression	1234	185	1419	0.016^*^	1.2 (1.05-1.53)
	31%	35.90%	31.20%		
Emergency medication use	1643	62	1705	<0.001^*^	0.2 (0.15-0.26)
	39.90%	11.70%	36.70%		
Physical hold	1064	66	1130	<0.001^*^	0.41 (0.31-0.53)
	25.90%	12.40%	24.30%		
Physical restraint	632	63	695	0.032^*^	0.74 (0.56-0.98)
	15.4%	11.80%	15%		

## Discussion

Consistent with previous reports, this study highlights the impact of COVID-related social distancing on the incidence of inpatient aggression. Studies have shown both increased and decreased rates of inpatient aggression during the COVID-19 pandemic [27-31]. One reason for this heterogeneity of results may be that many studies did not adjust for hospital bed occupancy in their analysis. The results of this study show that while the overall rate of inpatient aggression decreased during the COVID-19 pandemic, the census-adjusted rate of inpatient aggression did not change significantly compared to the pre-COVID group. One interpretation of this finding is that a decrease in hospital census was the main contributor to social distancing. Our results align with previous studies that show an association between bed occupancy rates and the incidence of inpatient aggression [1,26]. Results showed that the rate of recurrent aggression increased by about 5% in the post-COVID group. This is likely because individuals at lower risk of aggression were preferably discharged from the hospital to decrease the hospital census, and thus, individuals at higher risk of aggression continued to stay at the hospital and increased the risk of recurrent aggression. Concurrently, the impact of social distancing on inpatient aggression was observable in the form of reduced risk of seven-day and 14-day post-admission aggressive events. This is likely due to the isolation policy whereby all new admissions had to go through 14 days of isolation to prevent the spread of infection. There was also a decrease in emergency psychiatric medication usage as well as a decrease in utilization of physical hold and physical restraint for the management of aggressive events in the post-COVID group. These results may be due to the seclusion-like environment provided by the social distancing during the COVID-19 pandemic. Fear of contracting the coronavirus infection may also have caused hesitance in initiating safety measures that require staff-patient physical contact. Furthermore, social distancing measures may have provided more autonomy to nursing staff and a preference for non-pharmacological interventions. 

This study has several limitations. First, individual-level data was not considered in the analysis. Therefore, confounding patient factors, such as diagnosis and severity of psychopathology, were not accounted for. Data was also not available for other factors such as staff-patient ratio and the number of therapeutic activities in wards. It should also be noted that this study, being primarily a database review, introduces potential data entry errors. The COVID-19 pandemic was a unique time period and had a significant psychological impact on people in terms of personal safety. Therefore, although social distancing was a major theme, the observed changes in inpatient aggression may also be attributable to psychological factors, especially to fear of dying from COVID-19. Despite the methodological limitations, this study highlights the impact of social distancing on the incidence of inpatient aggression as well as on the utilization of restrictive measures such as medication administration and physical restraint.

## Conclusions

There was a decrease in the number of inpatient aggressive events after the implementation of social distancing measures during the COVID-19 pandemic. Consistent with previous reports, this study confirms the association between the hospital patient census and the incidence of inpatient aggression. More research is needed to explore whether social distancing (achieved either through a decrease in the number of patients or quarantining the new patients to a separate admission unit) can be utilized as a tool to decrease the incidence of inpatient aggression and the use of physical restraints.
